# APSP Journal of Case Reports Indexed with Pubmed and Pubmed Central

**Published:** 2013-01-01

**Authors:** Jamshed Akhtar

**Affiliations:** Department of Pediatric Surgery, National Institute of Child Health Karachi, Pakistan

It is a great pleasure for me to announce indexing of APSP Journal of Case Reports (AJCR) with Pubmed and Pubmed Central (PMC) [1]. This is a great achievement in a short span of time; less than three years to be precise. This could not have been achieved without support of our contributors. We hope to get the same cooperation in future as well.

Journals provide a portal through which researchers can publish their studies which contribute to evidence based practice. In the field of pediatric surgery, which is unique for myriad of anomalies that are not reported before, importance of reporting rare conditions benefit all those who are involved in the management of such cases. It also improves our understanding of various aspects of these conditions. AJCR has made immense contribution towards this end. This journal is dedicated to case reports only. The unique experiences of rare congenital malformations and unusual presentations of common conditions are available to readers in this journal. 

AJCR is open access. There are no publication charges for the authors. We encourage all, especially those from resource constraint countries, to report their experiences through AJCR. The Google analytics data shows that AJCR is accessed by readers from all over the world. Over the period of time, a large number of articles are received not only from South Asia but other parts of the world as well. The other unique feature of the journal is that it has only electronic version. The milestone of indexing with world renowned indexing bodies is already achieved and our next target is to get an impact factor. This may take at least two years as citation calculation process is based upon consecutive two years evaluation of published articles. 

The success we achieved can only be sustained through regular submission of quality articles. We hope to develop a long term partnership with researchers in this regard. We are also thankful to all the parents/family members who have allowed the authors, to use data of their loved ones, for better understanding of disease pattern that would benefit mankind.

## Footnotes

**Source of Support:** Nil

**Conflict of Interest:** None declared
